# Laparoscopic Treatment of Severe Gastroesophageal Reflux Disease (GERD) in Schaaf-Yang Syndrome: First Report of Toupet Fundoplication

**DOI:** 10.7759/cureus.87726

**Published:** 2025-07-11

**Authors:** Elisavet Kanna, Eleni Batsari, Zoi Lamprinou, Ioanna Argyri, Ioannis Skondras

**Affiliations:** 1 2nd Pediatric Surgery Department, Panagiotis & Aglaia Kyriakou Childrens' Hospital, Athens, GRC; 2 Undergraduate student of Nursing Department, National and Kapodistrian University of Athens, Athens, GRC; 3 Pediatric Gastroenterology, Panagiotis & Aglaia Kyriakou Childrens' Hospital, Athens, GRC

**Keywords:** laparoscopic fundoplication, laparoscopic toupet fundoplication, minimal invasive surgery, pediatric gastroesophageal reflux disease, schaaf-yang syndrome

## Abstract

Schaaf-Yang syndrome (SYS) is a rare genetic disorder marked by hypotonia, developmental delay, and feeding difficulties, including gastroesophageal reflux disease (GERD). We report the case of a 3-year-old boy with SYS who developed severe GERD and weight loss unresponsive to medical therapy. He underwent laparoscopic Toupet fundoplication with gastrostomy placement, resulting in complete symptom resolution and significant weight gain at one-year follow-up. To our knowledge, this is the first published report of Toupet fundoplication in a patient with SYS, highlighting its potential as a safe and effective surgical option for GERD management in this rare population.

## Introduction

Schaaf-Yang syndrome (SYS) is a rare, recently identified neurodevelopmental disorder caused by heterozygous truncating mutations in the *MAGEL2 *gene, a paternally expressed gene located in the Prader-Willi syndrome region (PWS) [1, 2]. One of the most prevalent postnatal features of SYS is developmental delay, accompanied by intellectual disability and severe hypotonia, which contributes to respiratory and feeding difficulties [3].

The existing international literature primarily focuses on the clinical phenotype and genetic background of individuals with SYS. Consequently, there is a paucity of knowledge regarding the management of syndrome symptoms, such as feeding difficulties, and more specifically, gastroesophageal reflux disease (GERD), which affects 50-60% of individuals with SYS [1,3].

This case report describes a 3-year-old child with SYS who underwent laparoscopic Toupet fundoplication to address severe gastroesophageal reflux. The procedure led to marked symptom relief and significant weight gain, with sustained improvement observed over a two-year follow-up. This case highlights the potential role of surgical management in improving outcomes for pediatric patients with SYS.

Toupet fundoplication was chosen due to its effectiveness in controlling reflux while reducing the risk of postoperative complications such as gas-bloat syndrome and dysphagia - concerns that are especially relevant in patients with hypotonia and neuromuscular impairment, as commonly seen in SYS.

## Case presentation

A 3-year-old male with SYS, diagnosed through whole-exome sequencing (WES), presented with characteristic clinical features, including psychomotor delay, unilateral cryptorchidism, muscle hypotonia, and gastroesophageal reflux. The diagnosis was made at 3 months of age following trio-based WES (patient and both parents), which identified a heterozygous truncating variant in the *MAGEL2 *gene of paternal origin. No methylation testing was performed.

The patient was born at term (38 weeks) via elective cesarean section due to a history of previous cesarean delivery, with Apgar scores of 8 and 9 at 1 and 5 minutes, respectively, and a birth weight of 3.270 kg. At 1.5 months of age, he remained in the neonatal intensive care unit (NICU) due to poor oral feeding, hypertonia, and difficulty managing oral secretions. Initial evaluations, including cranial imaging and metabolic screening, were unremarkable. He gradually improved and was able to feed orally, maintaining adequate weight gain until the age of 2 years.

Around that time, he began experiencing severe restlessness at bedtime and frequent regurgitation episodes, resulting in significant weight loss. Evaluation by otolaryngology and pediatric gastroenterology led to the initiation of lansoprazole therapy. Further investigations included 24-hour pHmetry, which confirmed pathological acid reflux, with prolonged periods of low pH recorded in the distal esophagus. Additional studies - including barium swallow and gastroscopy - revealed findings consistent with gastroesophageal reflux, such as mucosal irritation and delayed esophageal clearance.

Due to the severity of symptoms and rapid weight loss, the patient was evaluated by a multidisciplinary team including pediatric surgery, gastroenterology, nutrition, and anesthesiology. Preoperative assessment included nutritional evaluation, stabilization with nasogastric feeding, and optimization of gastroesophageal reflux symptoms with proton pump inhibitor therapy. The decision for surgery was based on persistent symptoms, failure to thrive, and radiological and pH-metric evidence of pathological reflux. At the time of intervention, the patient had lost approximately 1.5 kg over two months, corresponding to a significant drop in weight percentile.

A laparoscopic Toupet fundoplication (partial posterior 270° wrap of the gastric fundus around the lower esophagus) was performed to reinforce the lower esophageal sphincter and reduce reflux while preserving esophageal motility. In addition, a 12 Fr Stamm-type gastrostomy was placed to enable reliable enteral feeding and nutritional rehabilitation. Postoperative recovery was uneventful, and enteral feeds via gastrostomy were initiated gradually. The gastrostomy played a supportive role in stabilizing weight and preventing further nutritional deterioration.

No formal symptom scoring tools were used, but severity was assessed based on clinical presentation, regurgitation frequency, weight trend, and sleep disruption. Radiological findings and pHmetry supported the decision for surgery. No preoperative imaging is shown here, but it is available upon request.

**Figure 1:**
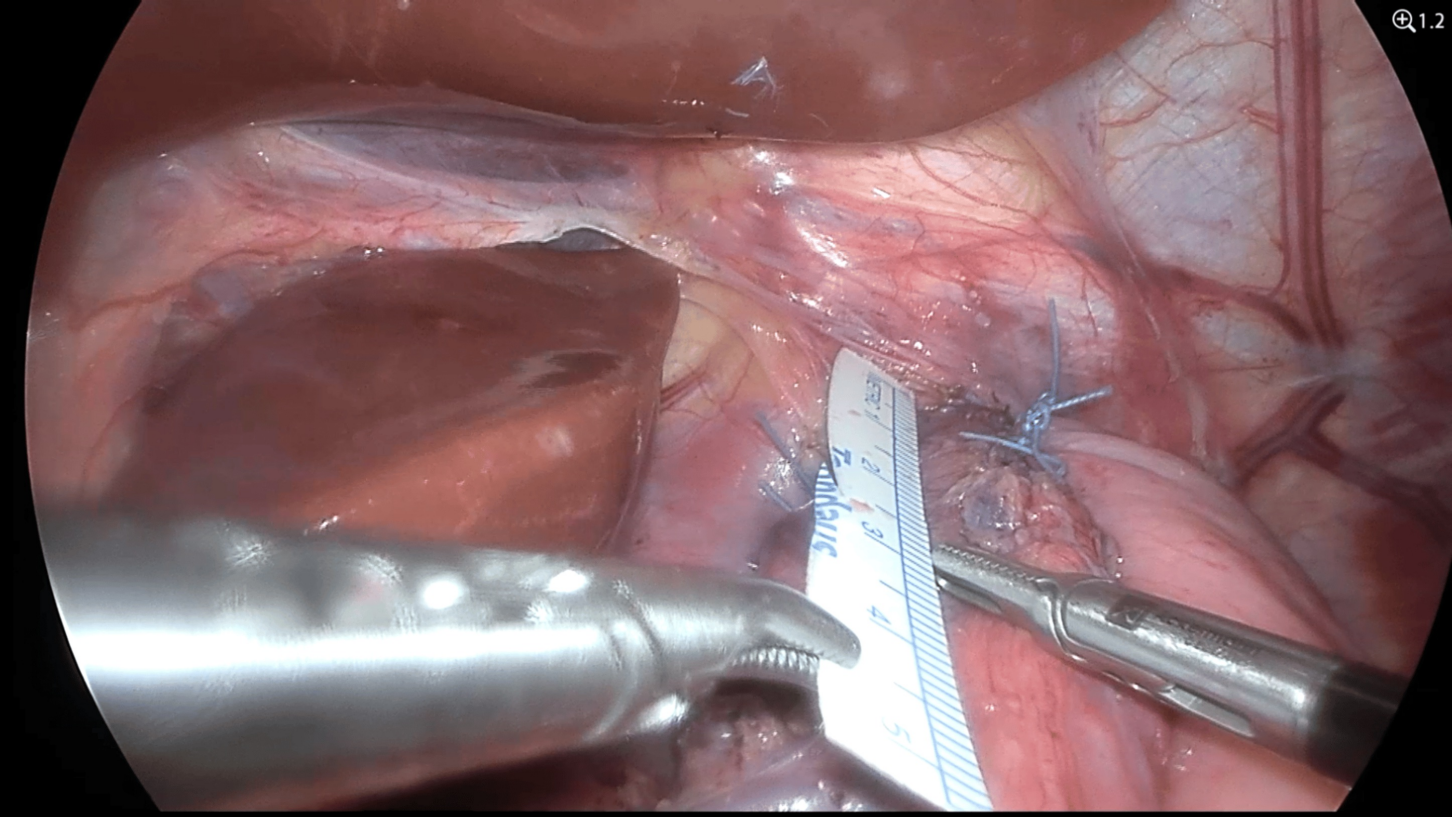
Intraoperative confirmation of ≥3 cm intra-abdominal esophageal length. Adequate esophageal mobilization is essential for tension-free wrap creation and long-term fundoplication success.

The surgical procedure began with the creation of pneumoperitoneum and the placement of standard laparoscopic ports. The hiatal dissection was carefully performed, with mobilization of the distal esophagus into the posterior mediastinum to achieve at least 3 cm of intra-abdominal esophagus, thereby ensuring sufficient length for a tension-free and effective fundoplication wrap. The short gastric vessels were divided to allow proper mobilization of the gastric fundus. The crural pillars were approximated with three sutures of non-absorbable polyester 2.0 to reinforce the hiatus and prevent future herniation. The fundoplication was constructed by securing the gastric fundus to the esophagus in a 270-degree posterior fashion using interrupted non-absorbable sutures. The wrap was additionally anchored to the right crus with a separate suture for enhanced stability (Figure 1).

Following the completion of the fundoplication, a Stamm-type gastrostomy was performed using a 12 French (Fr) gastrostomy tube, which was inserted percutaneously and secured to the anterior gastric wall. The gastrostomy site was further reinforced with additional sutures to reduce the risk of leakage or dislodgement. Importantly, the gastrostomy was also placed as a preventive measure to manage potential postoperative complications related to gas and air trapping - issues that have been described in patients with SYS, especially following Nissen fundoplication.

Hemostasis was carefully ensured throughout the procedure, and intraoperative endoscopic evaluation confirmed the correct positioning of the wrap and the gastrostomy tube. The procedure was completed without complications, with a total operative time of 150 minutes. The hemoglobin drop was minimal, recorded at only 1 g/dL.

One year postoperatively, the patient shows substantial improvement in quality of life, with complete resolution of previously reported symptoms, including nocturnal restlessness, frequent regurgitation, feeding difficulties, and weight loss. Lansoprazole therapy was discontinued six weeks after surgery, without recurrence of symptoms. The postoperative course was uneventful, with no infections or wound-related complications. The patient did not require ICU admission and was discharged on postoperative day 5. Over the first six months following surgery, the patient gained 2.1 kg, reflecting improved nutritional status and feeding tolerance. Importantly, no signs of hyperphagia or abnormal food-seeking behavior - features sometimes observed in later stages of SYS - were noted during follow-up. To date, there has been no recurrence of gastroesophageal symptoms, supporting the success of the surgical intervention.

## Discussion

SYS is a rare neurodevelopmental disorder characterized by a broad clinical spectrum, including intellectual disability, autism spectrum disorder, and seizures. Additional clinical features encompass hypotonia, respiratory and skeletal abnormalities, developmental and speech delay, sleep apnea, temperature instability, hypogonadism, and feeding disorders [4]. In its early stages, SYS exhibits significant clinical overlap with PWS. However, as patients age into childhood and adolescence, the syndrome evolves to present a more distinct clinical profile [5].

During childhood and adolescence, individuals with SYS often exhibit a progression of symptoms that distinguishes the condition from PWS. In SYS, skeletal abnormalities such as joint contractures and scoliosis tend to be more severe and commonly require orthopedic management, unlike in PWS, where significant joint deformities are uncommon. Additionally, neurological features - particularly seizures - are more frequently observed and often more severe in SYS, necessitating specialized neurological care. In contrast, seizures are relatively rare in individuals with PWS.

Intellectual disability and developmental delays are common to both disorders; however, individuals with SYS more often demonstrate pronounced impairments in executive function and adaptive behaviors compared to those with PWS. While this distinction highlights the broader clinical complexity of SYS, it also underscores the importance of addressing associated symptoms - such as GERD - which may be aggravated by neuromuscular and behavioral challenges. In this case, successful surgical management of GERD not only resolved gastrointestinal symptoms but also contributed to measurable improvements in nutritional status and quality of life, reinforcing the need for proactive, syndrome-specific interventions.

Endocrine dysfunctions, including hypogonadism and growth hormone deficiency, are common in both syndromes. However, obesity driven by hyperphagia is a hallmark of PWS, whereas SYS patients often continue to experience feeding difficulties and require gastrostomy placement, resulting in less concern for obesity. Sleep disturbances, particularly sleep apnea, are present in both disorders but tend to worsen with age in SYS due to persistent hypotonia, while in PWS, they are often exacerbated by obesity-related airway obstruction.

The gastrointestinal symptoms associated with SYS encompass swallowing difficulties during feeding, chronic constipation, and GERD, which is observed in 50-60% of affected individuals and is a matter of concern as a clinical entity in the present case. Feeding difficulties may resolve in the first year of life in up to 50% of affected individuals, but inadequate oral food intake remains a problem, resulting in the eventual need for gastrostomy placement, which may persist into childhood [4, 6].

Rare diseases such as SYS, for which only approximately 250 cases have been documented worldwide, pose significant challenges for clinicians in terms of their clinical spectrum and the management of associated symptoms. In this article, we present a case study of a patient diagnosed with SYS, with a particular focus on significant feeding difficulties related to GERD. These issues were managed surgically at the age of 3 years through a laparoscopic Toupet fundoplication, which resulted in complete resolution of symptoms.

A gastrostomy tube (12 Fr Stamm-type) had been inserted at the time of the surgical intervention to provide nutritional support during the perioperative period. The patient tolerated enteral feeds well, experienced no tube-related complications, and demonstrated a weight gain of 2.1 kg over the first six months postoperatively. Eventually, the patient transitioned back to full oral feeding and no longer required gastrostomy support.

A comprehensive literature search yielded no reports specifically describing laparoscopic Toupet fundoplication (LTF) as a treatment for a patient with SYS and GERD. One notable case involved an infant with SYS who presented with significant feeding difficulties and respiratory issues; at six weeks of age, the patient underwent gastrostomy tube placement combined with fundoplication and tracheostomy to address the high risk of aspiration and ongoing respiratory compromise. However, the specific type of fundoplication performed was not specified [7]. Additionally, a case was reported of a patient with SYS who underwent Nissen fundoplication at the age of 11 years, with feeding difficulties worsening by age 13 [4].

Recent comparative data from a prospective randomized study involving pediatric patients with GERD demonstrated that LTF is associated with a significantly lower incidence of early postoperative dysphagia compared to laparoscopic Nissen fundoplication (LNF) (P = 0.008), while maintaining similar efficacy in reflux symptom control [8]. Although the study did not specifically examine patients with SYS, these findings are relevant in the context of this case. Given the hypotonia and neuromuscular vulnerability commonly seen in SYS, the decision to perform an LTF - rather than a complete 360° wrap - was made to minimize the risk of postoperative dysphagia while still effectively managing reflux.

Although Dor fundoplication-a 180° anterior wrap-is sometimes considered for patients with severe esophageal dysmotility, it was not selected in this case. While it offers reduced risk of postoperative dysphagia, evidence suggests that its efficacy in preventing reflux recurrence is lower compared to the Toupet and Nissen techniques. A recent network meta-analysis of randomized controlled trials concluded that Toupet fundoplication offers an optimal balance between reflux control and minimizing postoperative complications, making it a suitable option for patients with neuromuscular vulnerability, such as those with SYS [9].

Nissen fundoplication is a commonly performed surgical procedure for GERD, but a subset of patients may continue to experience symptoms - such as persistent dysphagia, bloating, inability to belch, or recurrent reflux - despite a technically successful operation [10]. Several randomized controlled trials have compared LNF and LTF, showing that while both are similarly effective in reducing reflux and improving quality of life, LTF is associated with fewer postoperative complications, particularly dysphagia and gas-bloat syndrome [11].

The partial 270-degree posterior wrap used in LTF preserves more natural esophageal motility, whereas the complete 360-degree wrap in LNF can impair belching and increase the risk of postoperative dysphagia. As a result, LTF is often preferred in patients with esophageal dysmotility, including those with neuromuscular disorders such as SYS, while LNF may be more suitable for individuals with severe reflux and intact esophageal function. Although only a few reports of Nissen fundoplication in SYS exist, they raise concerns about increased postoperative complications, particularly gas-bloat syndrome and persistent dysphagia.

Despite these procedural differences, long-term outcomes and recurrence rates between LNF and LTF are generally comparable. However, due to its more favorable side-effect profile, LTF is increasingly considered a preferred alternative in selected patients. The clinical expectations of LTF and the postoperative outcomes in the present case are summarized in Table 1.

**Table 1:** Expected Outcomes of Laparoscopic Toupet Fundoplication (LTF) and Observed Postoperative Results in This Patient GERD: gastroesophageal reflux disease

Expected Outcome with LTF	Observed in This Patient
Lower risk of postoperative dysphagia	No dysphagia reported postoperatively
Preservation of belching ability	Belching preserved at follow-up
Lower incidence of gas-bloat syndrome	No signs of gas-bloat syndrome
Effective reflux control	Complete resolution of GERD symptoms
Suitable for esophageal dysmotility	Well tolerated in patient with Schaaf-Yang syndrome
Avoidance of feeding tube dependence	Transitioned to full oral feeding

This case highlights the successful use of LTF in treating GERD in a patient with SYS, a rare neurodevelopmental disorder. While the results are promising, this is a single-case report and should be interpreted with caution. Broader conclusions regarding surgical outcomes in SYS are limited by the lack of comparative or long-term data.

The identification of additional cases and the development of collective clinical experience are essential to establish evidence-based guidelines for managing GERD and other comorbidities in patients with SYS.

## Conclusions

SYS is an extremely rare genetic disorder characterized by a complex and evolving phenotype, including gastrointestinal manifestations such as feeding difficulties and gastroesophageal reflux. Many aspects of this syndrome remain poorly understood, highlighting the need for further clinical and research efforts. This case demonstrates that laparoscopic Toupet fundoplication can be an effective treatment option for GERD in patients with SYS, with resolution of symptoms and improved feeding outcomes.

The identification of additional cases and the systematic documentation of effective therapeutic strategies - such as LTF - will contribute to a more comprehensive understanding and improved management of SYS.
